# A Modified Bond-Associated Non-Ordinary State-Based Peridynamic Model for Impact Problems of Quasi-Brittle Materials

**DOI:** 10.3390/ma16114050

**Published:** 2023-05-29

**Authors:** Jing Zhang, Yaxun Liu, Xin Lai, Lisheng Liu, Hai Mei, Xiang Liu

**Affiliations:** 1 Hubei Key Laboratory of Theory and Application of Advanced Materials Mechanics, Wuhan University of Technology, Wuhan 430070, China; 2 Department of Engineering Structure and Mechanics, Wuhan University of Technology, Wuhan 430070, China

**Keywords:** peridynamics, impact response of ceramics, zero-energy mode, kinetic energy, bond-breaking criterion

## Abstract

In this work, we have developed a novel bond-associated non-ordinary state-based peridynamic (BA-NOSB PD) model for the numerical modeling and prediction of the impact response and fracture damage of quasi-brittle materials. First, the improved Johnson-Holmquist (JH2) constitutive relationship is implemented in the framework of BA-NOSB PD theory to describe the nonlinear material response, which also helps to eliminate the zero-energy mode. Afterwards, the volumetric strain in the equation of state is redefined by the introduction of the bond-associated deformation gradient, which can effectively improve the stability and accuracy of the material model. Then, a new general bond-breaking criterion is proposed in the BA-NOSB PD model, which is capable of covering various failure modes of quasi-brittle materials, including the tensile-shear failure that is not commonly considered in the literature. Subsequently, a practical bond-breaking strategy and its computational implementation are presented and discussed by means of energy convergence. Finally, the proposed model is verified by two benchmark numerical examples and demonstrated by the numerical simulation of edge-on impact and normal impact experiments on ceramics. The comparison between our results and references shows good capability and stability for impact problems of quasi-brittle materials. Numerical oscillations and unphysical deformation modes are effectively eliminated, showing strong robustness and bright prospects for relevant applications.

## 1. Introduction

In recent decades, ceramic materials have attracted great attention from researchers and engineers and have been extensively adopted for applications in the fields of aerospace, military equipment, biomedicine, and industrial manufacturing, due to its excellent performance, such as high hardness, high specific strength, high specific modulus, high temperature resistance and high abrasion resistance. In the initial stage, ceramics are applied in the field of armor protection with great success due to their excellent anti-penetration properties [1], which has great prospects. In military and protection applications, ceramics are often subjected to extreme loading conditions [2], such as shock waves from explosions or projectile penetration. Therefore, extensive research and testing have been devoted to the understanding of the fundamental nature and mechanism of the impact response and anti-penetration behavior of ceramic materials. Various approaches and numerical simulations have been carried out to study the damage and fracture of ceramics [3,4,5], including but not limited to the finite element method (FEM), cohesive zone model (CZM), and extended finite element method (XFEM). However, due to the mesh dependence, the damage or fracture of materials leads to grid distortion, and the calculation cannot continue in mesh-based approaches. On the other hand, the cohesive zone model only allows cracks to propagate along element boundaries, which may not be able to accurately describe the crack path. In XFEM, although cracks can propagate along any direction, the introduction of additional degrees of freedom brings considerable computational complexity. Damage or fracture will result in discontinuity of the displacement field, which creates inherent limitations for the above numerical simulation methods based on continuum mechanics. To date, it is still challenging for numerical approaches to give a precise prediction of the complex fracture and damage patterns on ceramics.

To naturally simulate the discontinuous behavior of materials such as damage and cracking, Silling [6] proposed the peridynamic (PD) theory with nonlocal interactions as the core. This theory is also called bond-based PD (BB PD). In the PD theory, a body is described by a set of discretized material points with mass and other physical properties. The description of macroscopic material properties is formed by nonlocal bonding and interaction between material points. The damage of materials is part of PD theory, which is described by breaking bonds and truncating interactions between material points. PD is a theory of nonlocal continuum mechanics, which uses integral equations instead of integral–differential equations and does not require the displacement field to satisfy the requirement of continuous derivability. Therefore, the PD theory is well suited to modeling discontinuity problems without the difficulties encountered by classical local theory. Further, Silling [7] successfully proved the ability of PD theory to model complex fracture problems using the Kalthoff–Winkler experiment. In addition, Silling and Askari [8] developed the PD prototype microelastic brittle (PMB) material model and its numerical implementation technology, and derived the critical stretch formulation. The BB PD theory proposed earlier does not include stress and strain measurements, encountered the restriction of fixed Poisson’s ratio, and cannot describe the shear deformation of materials, which had certain limitations. Hence, Silling et al. [9] proposed the state-based PD (SB PD) theory, which extends the PD theory and the material responses it can describe. The theory can be divided into the ordinary state-based PD (OSB PD) and the non-ordinary state-based PD (NOSB PD), depending on the form of interactions.

The PD theory has significant advantages in modeling discontinuity problems, and is widely used in simulating material damage, cracks, and fractures. Some promising applications of the PD theory in damage prediction and fracture modeling are outlined below. Ha and Bobaru [10,11] reproduced the crack growth and fracture behavior of glass materials in dynamic tensile experiments. Zhu and Ni [12] proposed the bond rotation effect in BB PD, which removed the limitation of Poisson’s ratio. Chu et al. [13] presented a rate-dependent BB PD model to simulate the anti-penetration behavior of ceramics. Liu et al. [14] proposed a comprehensive tensile-shear and compressive-shear failure criterion to evaluate the impact damage and fracture characteristics of ceramics. Erdogan and Oterkus [15] proposed an OSB PD model based on the Mises yield criterion. Zhang and Qiao [16] established a new bond-breaking criterion based on the critical relative rotation angle. Foster et al. [17] implemented an elastic viscoplastic constitutive relationship in NOSB PD and reproduced the Taylor impact test of 6061-T6 aluminum. O’Grady and Foster [18] developed a Euler–Bernoulli beam model. Lai et al. [19,20] implemented the Drucker–Prager and JH2 constitutive models in NOSB PD to model dynamic brittle fracture of quasi-brittle materials. Wu et al. [21] introduced the Holmquist–Johnson–Cook (HJC) constitutive theory to analyze the dynamic mechanical behavior of concrete during impact. Wang et al. [22] studied the thermo-viscoplastic responses of metals under impact loads. Zhu and Zhao [23] simulated single-hole rock blasting with the JH2 damage and tensile failure models. Yang et al. [24] modeled the impact spalling of concrete based on the BA-NOSB PD model for quasi-brittle materials. Li et al. [25] presented a PD model using the Mindlin–Reissner shell theory and simulated the brittle fracture of thin shells.

Although the capability of the NOSB PD model incorporating constitutive models of classical continuum mechanics to simulate complex material responses has been demonstrated, there are still urgent problems to be addressed. The zero-energy mode is one of the major challenges in the NOSB PD theory, which leads to numerical oscillations, affects calculation accuracy, and even produces incorrect calculation results. To date, a number of control schemes and stabilization strategies have been proposed. For example, a stable supplemental force state [26,27,28,29] has been added to the original force vector state to control the zero-energy mode. Yaghoobi and Chorzepa [30] suppressed numerical oscillations by introducing a higher-order version of the conventional deformation gradient. The stress-point method [31] and the higher-order stress-point method [32] were also developed to remove numerical oscillations. In particular, zero-energy mode oscillations are believed to be caused by non-unique mapping relationships from the deformation state to the force vector state, due to the conventional deformation gradient. Therefore, Chen [33] proposed the bond-associated deformation gradient to suppress or eliminate numerical oscillations. The BA-NOSB PD model has also been proved to meet the requirements for stability and convergence [34]. Gu et al. [35] further studied and discussed the possible causes for numerical oscillations in the model. On the other hand, the treatment of broken bonds is also a key problem that deserves attention and needs to be addressed. For the SB PD theory, the force vector state at a material point depends on the deformation behavior of all bonds in its family over time. Therefore, the treatment of broken bonds has a crucial effect on the calculation of deformation states for unbroken bonds. This further affects the stability of the material model, and needs to be taken into account. Silling [27] and Li et al. [36] introduced damage to the influence function so that a broken bond was no longer included in subsequent calculations. In order to make better use of NOSB PD to simulate complex material responses, the material model stability and the treatment of broken bonds will be part of our work.

Following the above introduction, in Section 2, we first briefly review the original PD models for quasi-brittle materials. After that, a modified BA-NOSB PD model is established, and its constitutive update scheme is presented. Then, the other four zero-energy mode control schemes are introduced. Subsequently, the numerical discretization and implementation used in this paper are discussed. In Section 3, the modified model is verified using two benchmark examples, and numerical simulations of edge impact and normal impact experiments on ceramics are carried out. Finally, the conclusions of this work are summarized in Section 4.

## 2. Methodology

### 2.1. Brief Review of Peridynamic Models for Quasi-Brittle Materials

To date, a variety of theoretical models [13,14,19,20,21,23,24,37,38,39] have been developed for damage prediction and fracture modeling of quasi-brittle materials. In this section, two PD models for quasi-brittle materials [20,24] are briefly reviewed. First, the original and bond-associated NOSB PD theory and their basic formulas are presented in Section 2.1.1 and Section 2.1.2, respectively. Then, the JH2 material model used for the constitutive update of the above two PD theories is reviewed in Section 2.1.3.

#### 2.1.1. Original Non-Ordinary State-Based Peridynamics

In the PD theory, a body is modeled by a finite number of discretized material points. Further, the material point X interacts only with the material point $X^{\prime }$ within a limited range, and the limited range of influence is called horizon δ. The material point $X^{\prime }$ is called the neighbor of its center point X, and all the neighbors of the material point X form the family $H_{X}$, i.e., $H_{X}=H\left(X,\delta \right)=\left\{X^{\prime }\in B:\;\left\{\left\|X^{\prime }-X\right\|\leq \delta \right\}\right\}$. In the reference configuration, the reference position state is defined as

(1) $$\underline{X}\langle \xi \rangle =X^{\prime }-X=\xi .$$

In the current configuration, the relative displacement state and the deformation state are defined as

(2) $$\underline{U}\langle \xi \rangle =u^{\prime }-u=\eta ,$$

(3) $$\underline{Y}\langle \xi \rangle =y^{\prime }-y=\xi +\eta ,$$

where y and $y^{\prime }$ are the spatial positions of the two material points, η is a relative displacement between them, and ξ is called a bond.

The NOSB PD theory [9] is a generalization of BB PD, which provides generality in modeling material responses, such as volumetric strain or shear deformation. In NOSB PD, the equation of motion for the material point X at the instant of t can be expressed as

(4) $$\rho_{0}(X)\ddot{u}\left(X,t\right)=\int_{H_{X}}\left[\underline{T}\left(X,t\right)\langle \xi \rangle -\underline{T}\left(X^{\prime },t\right)\langle -\xi \rangle \right]dV_{X^{\prime }}+b\left(X,t\right),$$

in which $\rho_{0}(X)$ represents the mass density of materials, $\ddot{u}\left(X,t\right)$ represents the acceleration, $b\left(X,t\right)$ represents the external body force density, and $\underline{T}\left(X,t\right)$ represents the force vector state. In particular, the force state $\underline{T}\left(X,t\right)$ is a function of the deformation state $\underline{Y}$.

In NOSB PD, the material response of a material point will depend on the deformation behavior of all bonds within its horizon. The mapping relationship between the deformation state and the force vector state is the constitutive model of materials in the PD framework. The force density vector at the material point X can be written as

(5) $$t\left(X,t\right)=\underline{T}\left(X,t\right)\langle \xi \rangle =\underline{\omega }\langle \left\|\xi \right\|\rangle PK^{-1}\underline{X}\langle \xi \rangle ,$$

in which $\underline{\omega }\langle \left\|\xi \right\|\rangle$ is the influence function on a bond.

The shape tensor K is defined as

(6) $$K=\int_{H_{X}}\underline{\omega }\langle \left\|\xi \right\|\rangle \underline{X}\langle \xi \rangle ⊗\underline{X}\langle \xi \rangle dV_{X^{\prime }}$$

The first Piola–Kirchhoff stress tensor can be calculated by classical constitutive models, as

(7) $$P=J\sigma F^{-T},$$

in which $J=\det\left(F\right)$, T is the matrix transposition operator, and σ is the Cauchy stress. The approximate nonlocal deformation gradient tensor F is defined as

(8) $$F=\left(\int_{H_{X}}\underline{\omega }\langle \left\|\xi \right\|\rangle \underline{Y}\langle \xi \rangle ⊗\underline{X}\langle \xi \rangle dV_{X^{\prime }}\right)K^{-1}.$$

#### 2.1.2. Bond-Associated Non-Ordinary State-Based Peridynamics

The zero-energy mode can lead to instability of the material model, which is manifested in numerical oscillations of solutions or completely erroneous calculation results. To eliminate the numerical oscillations caused by the zero-energy mode, Chen [33] proposed the BA-NOSB PD theory and proved the convergence and stability of the PD model [34].

**Figure 1:**
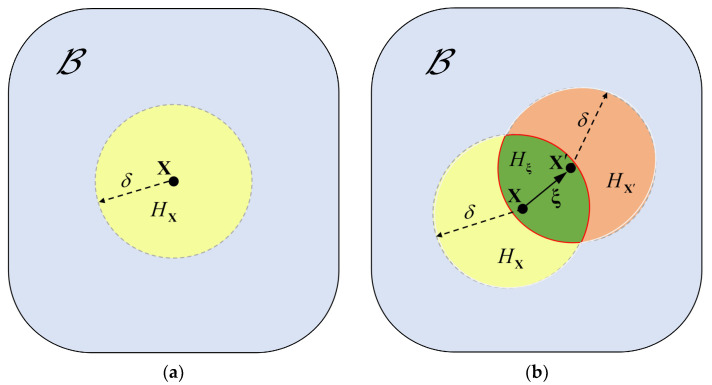
Definitions of different horizons. (**a**) Point-associated horizons; (**b**) bond-associated horizons.

In the original NOSB PD theory, the point-associated deformation gradient results in a non-unique mapping relationship from the deformation vector state to the force vector state. The definition of “bond-associated horizon” is presented to meet the requirement of the unique mapping relationship. The bond-associated horizon is defined as $H_{\xi }=H_{X}\cap H_{X^{\prime }}$, as shown in Figure 1. In BA-NOSB PD, an individual bond corresponds to a unique deformation gradient tensor. Thus, the bond-associated deformation gradient tensor with respect to the bond ξ at the material point X is defined as

(9) $$F_{b}=\left(\int_{H_{\xi }}\underline{\omega }\langle \left\|\xi \right\|\rangle \underline{Y}\langle \xi \rangle ⊗\underline{X}\langle \xi \rangle dV_{X^{\prime }}\right)K_{b}^{-1},$$

where the subscript b indicates that the physical quantity is bond-associated. The bond-associated deformation gradients ensure the desired unique mapping relationship, and the bond-associated shape tensor can be defined as

(10) $$K_{b}=\int_{H_{\xi }}\underline{\omega }\langle \left\|\xi \right\|\rangle \underline{X}\langle \xi \rangle ⊗\underline{X}\langle \xi \rangle dV_{X^{\prime }}.$$

The nonlocal deformation gradient tensor F is redefined based on Equation (9) by weighted averaging as

(11) $$F=\frac{\sum_{n=1}^{N_{b}}\underline{\omega }\langle \left\|\xi^{n}\right\|\rangle F_{b}^{n}}{\sum_{n=1}^{N_{b}}\underline{\omega }\langle \left\|\xi^{n}\right\|\rangle }.$$

where $N_{b}$ represents the number of bond-associated neighbors at the material point X.

The bond-associated force density vector is recalculated as

(12) $$t_{b}\left(X,t\right)=\underline{T_{b}}\left(X,t\right)\langle \xi \rangle =\frac{\int_{H_{\xi }}1dV_{X^{\prime }}}{\int_{H_{X}}1dV_{X^{\prime }}}\underline{\omega }\langle \left\|\xi \right\|\rangle P_{b}K_{b}^{-1}\underline{X}\langle \xi \rangle ,$$

The bond-associated first Piola–Kirchhoff stress tensor is defined as

(13) $$P_{b}=J_{b}\sigma_{b}F_{b}^{-T},$$

in which $J_{b}=\det\left(F_{b}\right)$, and $\sigma_{b}$ is the bond-associated Cauchy stress tensor.

According to Gu’s work [35], the bond-associated deformation gradient not only satisfies a unique mapping relation from the deformation state to the force vector state, but also satisfies the kinematic constraints of the bonds, as follows:

(14) $$\left\{\begin{array}{l} \xi +\eta =F_{b}\left(X,\xi \right)\xi \\ \xi +\eta =F_{b}\left(X^{\prime },-\xi \right)\xi \end{array}\right..$$

Moreover, both the bond-associated shape tensors and the bond-associated deformation gradient tensors satisfy the symmetry, i.e., $K_{b}\left(X,\xi \right)=K_{b}\left(X^{\prime },-\xi \right)$ and $F_{b}\left(X,\xi \right)=F_{b}\left(X^{\prime },-\xi \right)$. The bond-associated first Piola–Kirchhoff stress tensors satisfies symmetry as well:

(15) $$P_{b}\left(X,\xi \right)=P_{b}\left(X^{\prime },-\xi \right).$$

**Figure 2:**
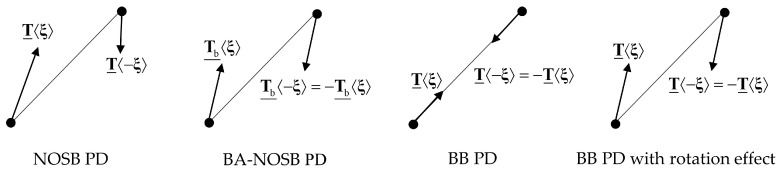
Force vector state for PD models.

As shown in Figure 2, similar to the BB PD model with rotation effect [12], the two bond-associated force density vectors in a bond are also equal in magnitude and opposite in direction, but not parallel to the bond, which can be written as

(16) $$\underline{T_{b}}\left(X,t\right)\langle \xi \rangle =-\underline{T_{b}}\left(X^{\prime },t\right)\langle -\xi \rangle .$$

Finally, in BA-NOSB PD, the equation of motion for the material point X at time t can be expressed as

(17) $$\rho_{0}(X)\ddot{u}\left(X,t\right)=\int_{H_{X}}\left[\underline{T_{b}}\left(X,t\right)\langle \xi \rangle -\underline{T_{b}}\left(X^{\prime },t\right)\langle -\xi \rangle \right]dV_{X^{\prime }}+b\left(X,t\right).$$

#### 2.1.3. JH2 Constitutive Model

The quasi-brittle materials also have important engineering applications, such as armored ceramics, bulletproof glass, concrete dams, and rock materials. The mechanical behavior and material response of these materials in different applications is an important basis for evaluating and measuring their safety in use and working life. To date, some constitutive models [40,41,42,43,44] describing quasi-brittle materials have been developed and used. The original Johnson–Holmquist (JH1) model [45] does not allow gradual softening, may damage self-healing, and is too sensitive to material model parameters. The JH2 model [46] is an improved version of the JH1 model, which is a widely used constitutive model for brittle or quasi-brittle materials, including ceramics, concrete, and rocks.

In the JH2 constitutive model, the equivalent stress is normalized as

(18) $$\sigma^{*}=\sigma_{i}^{*}-D_{\mathrm{JH}2}\left(\sigma_{i}^{*}-\sigma_{f}^{*}\right),$$

in which $\sigma_{i}^{*}$ is the normalized intact equivalent stress of materials, $\sigma_{f}^{*}$ is the normalized fracture equivalent stress of materials, and $D_{\mathrm{JH}2}$ is dimensionless damage ($0\leq D_{\mathrm{JH}2}\leq 1.0$).

The true equivalent stress can be normalized as

(19) $$\sigma^{*}=\sigma /\sigma_{\mathrm{HEL}},$$

in which σ represents the true equivalent stress, $\sigma_{\mathrm{HEL}}$ represents the equivalent stress at HEL, and HEL represents the Hugoniot elastic limit.

The normalized intact equivalent strength of materials is expressed as

(20) $$\sigma_{i}^{*}=A{\left(P^{*}+T^{*}\right)}^{N}\left(1+C\cdot \ln{\dot{\varepsilon }}^{*}\right).$$

The normalized fracture equivalent strength of materials is expressed as

(21) $$\sigma_{f}^{*}=B{\left(P^{*}\right)}^{M}\left(1+C\cdot \ln{\dot{\varepsilon }}^{*}\right).$$

Also, the fracture equivalent strength of the material should satisfy $\sigma_{f}^{*}\leq \mathrm{SFMAX}$. Moreover, A, B, C, M, N, and SFMAX are material parameters. ${\dot{\varepsilon }}^{*}=\dot{\varepsilon }/{\dot{\varepsilon }}_{0}$ is the dimensionless strain rate, in which $\dot{\varepsilon }$ is the true strain rate and ${\dot{\varepsilon }}_{0}=1.0{\;s}^{-1}$.

The normalized hydrostatic pressure is expressed as

(22) $$P^{*}=P/P_{\mathrm{HEL}},$$

where P is the true pressure, and $P_{\mathrm{HEL}}$ is the pressure at HEL.

The maximum tensile hydrostatic pressure is normalized to

(23) $$T^{*}=T/P_{\mathrm{HEL}},$$

where T is the maximum hydrostatic tension that can be sustained.

Similar to the JH1 model [45], the damage in the JH2 model is accumulated as

(24) $$D_{\mathrm{JH}2}=\sum \Delta \varepsilon^{P}/\varepsilon_{f}^{P},$$

in which $\Delta \varepsilon^{P}$ is the plastic strain accumulated under the pressure P. The fracture plastic strain can be expressed as

(25) $$\varepsilon_{f}^{P}=D_{1}{\left(P^{*}+T^{*}\right)}^{D_{2}},$$

in which $D_{1}$ and $D_{2}$ are material parameters. In addition, the material can no longer withstand any plastic strain when $P^{*}=-T^{*}$, but $\varepsilon_{f}^{P}$ can increase with the increase in $P^{*}$.

Before materials fracture ($D_{\mathrm{JH}2}=0$), the energy loss can be negligible. At this moment, the hydrostatic pressure is

(26) $$P=\left\{\begin{array}{cc} K_{1}\mu +K_{2}\mu^{2}+K_{3}\mu^{3}, & \mathrm{If}\;\mu \geq 0\\ K_{1}\mu , & \mathrm{otherwise} \end{array}\right..$$

After fractured ($D_{\mathrm{JH}2}>0$), damage begins to accumulate. As the volumetric strain increases, volume bulking can occur. Due to energy loss, incremental pressure ΔP is added to the pressure P as

(27) $$P=K_{1}\mu +K_{2}\mu^{2}+K_{3}\mu^{3}+\Delta P,$$

in which $K_{1}$, $K_{2}$, and $K_{3}$ are material parameters, and μ is the volumetric strain. The material is in tension when μ≥0, otherwise it is in compression.

The elastic internal energy can be written as

(28) $$U=\sigma^{2}/6G,$$

where σ is the equivalent stress, and G is the shear modulus.

The incremental energy loss can be calculated as

(29) $$\Delta U=U_{D_{\mathrm{JH}2}\left(t-\Delta t\right)}-U_{D_{\mathrm{JH}2}\left(t\right)},$$

where $U_{D_{\mathrm{JH}2}\left(t-\Delta t\right)}$ and $U_{D_{\mathrm{JH}2}\left(t\right)}$ are the elastic internal energies calculated from Equation (28) for the corresponding time step.

The updated incremental pressure ΔP is calculated as

(30) $$\Delta P_{t}=-K_{1}\mu_{t}+\sqrt{{\left(K_{1}\mu_{t}+\Delta P_{t-\Delta t}\right)}^{2}+2\beta K_{1}\Delta U},$$

in which β is the material parameter, and ΔP is the incremental pressure.

### 2.2. Modified Bond-Associated Non-Ordinary State-Based Peridynamic Model

In this section, the original BA-NOSB PD model based on the JH2 constitutive relationship is modified to more accurately model the impact problems of quasi-brittle materials. First, the concept of “bond-associated volumetric strain” and its new calculation scheme are proposed via the introduction of the bond-associated deformation gradient. Then, a general tensile-shear coupling bond-breaking criterion is proposed in the framework of the BA-NOSB PD theory. Finally, a new treatment strategy for broken bonds is also given.

#### 2.2.1. Bond-Associated Volumetric Strain

It is worth noting that all physical quantities are bond-associated within the BA-NOSB PD theory. Some improvements are proposed in this section to implement a fully bond-associated JH2 constitutive model.

The deformation of an object under the action of an external force can generally be classified as volume change and shape change. In the theory of plasticity, it is generally considered that the volume change is caused by spherical stresses, while the shape change is caused by deviatoric stresses. Therefore, the stress state at a point can be decomposed into a spherical stress state and a deviatoric stress state. In addition, the stress state at the point is determined by the stress tensor σ, which can be expressed as

(31) σ=PI+S,

in which PI indicates the spherical stress tensor, P is the pressure, I is a second-order identity tensor, and S indicates the deviatoric stress tensor.

In the framework of BA-NOSB PD theory, the above equation can be rewritten as

(32) $$\sigma_{b}=P_{b}I+S_{b},$$

where $S_{b}$ represents the bond-associated deviatoric stress tensor, which is given in Section 2.3. According to Equation (26), the bond-associated hydrostatic pressure $P_{b}$ can be calculated as

(33) $$P_{b}=\left\{\begin{array}{cc} K_{1}\mu_{b}+K_{2}\mu_{b}^{2}+K_{3}\mu_{b}^{3}, & \mathrm{If}\;\mu_{b}\geq 0\\ K_{1}\mu_{b}, & \mathrm{otherwise} \end{array}\right..$$

As seen in Equation (33), the bond-associated hydrostatic pressure $P_{b}$ depends on the bond-associated volumetric strain $\mu_{b}$. In the original JH2 constitutive model [45], the volumetric strain μ in the equation of state is defined as

(34) $$\mu =\rho /\rho_{0}-1,$$

where $\rho_{0}$ is the initial mass density, and ρ is the current mass density.

The volumetric strain μ in Equation (34) depends only on the mass density of a point, and can also be expressed as

(35) $$\mu =\mu_{p}=dV/dv-1,$$

in which the subscript b indicates that the physical quantity is point-associated, dV is the reference volume element, and dv is the current volume element.

Referring to classical continuum mechanics, and according to the third invariant of the bond-associated deformation gradient tensor $F_{b}$ in Section 2.1.2, $J_{b}=\det\left(F_{b}\right)$, the transformation relationship between volume elements under different configurations can be expressed as

(36) $$dV=J_{b}dv.$$

The equation above holds for any shaped volume element since it can be approximated by an infinite number of parallel hexahedra. Substituting Equation (36) into Equation (35), the bond-associated volumetric strain $\mu_{b}$ can be defined as

(37) $$\mu =\mu_{b}=1/J_{b}-1.$$

Obviously, $\mu_{p}$ is a point-associated nonlocal physical quantity, while $\mu_{b}$ is a bond-associated nonlocal physical quantity. Without losing generality, in addition to the JH2 model [45], the defined bond-associated volumetric strain $\mu_{b}$ can also be used in other constitutive models [46,47,48] implemented in the framework of the BA-NOSB PD theory. Further details of the constitutive update using the JH2 model will be given in Section 2.3.

#### 2.2.2. Bond-Breaking Criterion

If the external force exceeds the fracture strength of materials, local damage, cracks or fractures will occur. The PD can naturally model discontinuous behavior such as damage or cracks, and damage is also incorporated into its constitutive model. In the PD theory, the bonds are carriers that transmit non-local interactions, while damage is defined by breaking bonds. In addition, broken bonds will no longer transmit PD interaction. Accurately assessing and describing the discontinuity behavior of materials usually requires a robust bond-breaking criterion.

**Figure 3:**
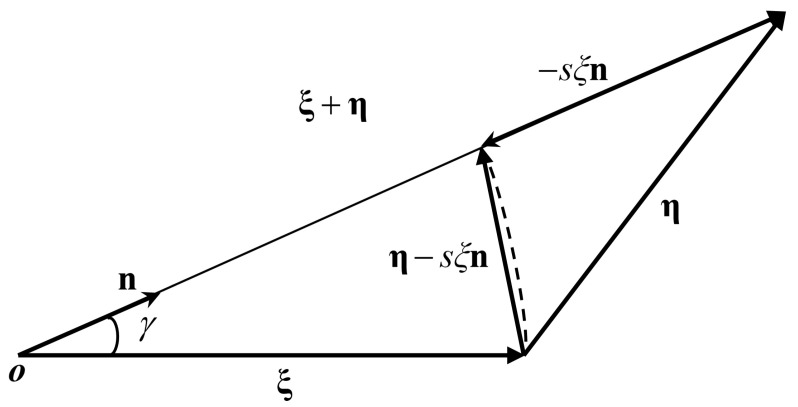
Schematic diagram of the relative rotation angle [14].

In most engineering problems, materials usually suffer shear failure, not just tensile and compressive failure. According to Equation (16), the bond-associated force density vector can describe not only tensile-compressive deformation, but also shear deformation. However, the critical stretch criterion [8] proposed earlier cannot evaluate material damage caused by shear deformation. Therefore, the assessment of shear damage needs to be refined in BA-NOSB PD. Based on the comprehensive failure criterion proposed by Liu et al. [14], a new general tensile-shear coupling bond-breaking criterion is proposed and implemented in the framework of BA-NOSB PD theory. As shown in Figure 3, under the condition of small deformation, the relative rotation angle vector [12] is approximated as

(38) $$\gamma =\frac{\eta -s\xi n}{\xi }$$

where $n=\left(\xi +\eta \right)/\left\|\xi +\eta \right\|$ is an identity direction vector of the bond ξ in the current configuration, and $\xi =\left\|\xi \right\|$.

The bond stretch in PD is defined as

(39) $$s\left(\xi ,\eta \right)=\frac{\left\|\xi +\eta \right\|-\left\|\xi \right\|}{\left\|\xi \right\|}.$$

Similarly, the bond-breaking threshold used to assess the tensile-shear failure of materials in this work is defined by the dimensionless quantity λ, which can be expressed as

(40) $$\lambda =\sqrt{\alpha_{0}{\left(\frac{s}{s_{0}}\right)}^{2}+\beta_{0}{\left(\frac{\gamma }{\gamma_{0}}\right)}^{2}},$$

in which $s_{0}$ is the critical stretch, $\gamma_{0}$ is the critical relative rotation angle, and $\gamma =\left\|\gamma \right\|$ is the relative rotation angle. The difference between the anti-tensile capability and anti-shear capability of materials is considered in this work by defining the anti-tensile weight $\alpha_{0}$ and anti-shear weight $\beta_{0}$ respectively, as follows:

(41) $$\left\{\begin{array}{l} \alpha_{0}=\frac{s_{0}}{s_{0}+\gamma_{0}}\\ \beta_{0}=\frac{\gamma_{0}}{s_{0}+\gamma_{0}} \end{array}\right..$$

In particular, the tensile-shear coupling bond-breaking criterion degrades to the critical stretch criterion when the anti-tensile weight and anti-shear weight are taken as $\alpha_{0}=1$ and $\beta_{0}=0$, respectively.

Further, the critical stretch $s_{0}$ and the critical relative rotation angle $\gamma_{0}$ are defined by the fracture energy of materials. In the PD theory, the energy release rate $G_{0}$ [8] and the shear fracture energy $G_{s}$ [14] are respectively expressed as

(42) $$\left\{\begin{array}{c} G_{0}=\frac{\pi cs_{0}^{2}\delta^{5}}{10}\\ G_{S}=\frac{\pi c\gamma_{0}^{2}\delta^{5}}{10} \end{array}\right..$$

For the three-dimensional case, the critical stretch is calculated as

(43) $$s_{0}=\sqrt{5G_{0}/\left(9K\delta \right)},$$

where K represents the bulk modulus.

Similar to the derivation of the critical stretch $s_{0}$ by Silling and Askari [8], substituting the bond constant $c=18K/\pi \delta^{4}$ of the PMB model into Equation (42), the critical relative rotation angle $\gamma_{0}$ is expressed as

(44) $$\gamma_{0}=\sqrt{5G_{s}/\left(9K\delta \right)}.$$

In the PD theory, the local damage of the material point X at the instant of t can be defined as

(45) $$\varphi \left(X,t\right)=1-\frac{\int_{H_{X}}\mu \left(\xi ,t\right)dV_{X^{\prime }}}{\int_{H_{X}}dV_{X^{\prime }}},$$

where the scalar function $\mu \left(\xi ,t\right)$ is

(46) $$\mu \left(\xi ,t\right)=\left\{\begin{array}{cc} 1, & \lambda <1.0\\ 0, & \lambda \geq 1.0 \end{array}\right.$$

#### 2.2.3. Treatment Strategy of Broken Bonds

Unlike BB PD theory, the force density vectors at a material point depend on the deformation behavior of all bonds in SB PD. Therefore, the description of broken bonds in the state-based theory is very important, since it will affect the calculation update of the force vector state for unbroken bonds. The following two treatment strategies are given for a broken bond, which will be compared and discussed in the subsequent Section 3.2. If the bond ξ breaks, in each subsequent time-integral cycle, the relative position vector between two material points in the current configuration is considered as

(47) $${\underline{Y}}_{B}\langle \xi \rangle =\xi ,$$

(48) $${\underline{Y}}_{B}\langle \xi \rangle =\xi +\eta_{B},$$

in which the subscript B indicates that the bond has been broken, and $\eta_{B}$ is the relative displacement vector between two material points when the bond ξ breaks.

### 2.3. Constitutive Update Scheme

In this section, the constitutive update scheme using the JH2 model is given in the framework of the BA-NOSB PD theory. First, based on the bond-associated deformation gradient tensor $F_{b}$, the bond-associated spatial velocity gradient tensor $L_{b}$ can be calculated as

(49) $$L_{b}={\dot{F}}_{b}F_{b}^{-1},$$

where ${\dot{F}}_{b}$ is the material derivative of the deformation gradient $F_{b}$, which can be calculated as

(50) $${\dot{F}}_{b}=\int_{H_{\xi }}\underline{\omega }\langle \left\|\xi \right\|\rangle \dot{\underline{Y}}\langle \xi \rangle ⊗\underline{X}\langle \xi \rangle dV_{X^{\prime }}K_{b}^{-1}.$$

The spatial velocity gradient $L_{b}$ is decomposed into a bond-associated deformation rate tensor $D_{b}$ and a bond-associated rotation rate tensor $W_{b}$:

(51) $$\begin{array}{ccc} D_{b}=\frac{1}{2}\left(L_{b}+L_{b}^{T}\right) & \mathrm{and} & W_{b}=\frac{1}{2}\left(L_{b}-L_{b}^{T}\right) \end{array}.$$

To ensure that the resulting constitutive relationship satisfies the principle of coordinate invariance, the algorithm proposed by Rubinstein and Atluri [49] is used to update the bond-associated Cauchy stress $\sigma_{b}$. Before this, the bond-associated unrotated deformation rate of the tensor $d_{b}$ can be expressed as

(52) $$d_{b}={\left(R_{b}^{t}\right)}^{T}D_{b}R_{b}^{t},$$

where $R_{b}^{t}$ is the bond-associated orthogonal rotation tensor that describes the rigid-body rotation, which can be calculated as

(53) $$R_{b}^{t}=\left[I+\frac{\sin\left(\Delta t\Omega \right)}{\Omega }\Omega -\frac{1-\cos\left(\Delta t\Omega \right)}{\Omega^{2}}\Omega^{2}\right]R_{b}^{t-\Delta t},$$

in which the rigid-body rotation rate tensor Ω can be calculated as

(54) $$\left\{\begin{array}{c} z_{i}=e_{ikj}{\left(D_{b}\right)}_{jm}{\left(V_{b}\right)}_{mk}\\ w_{i}=-\frac{1}{2}e_{ijk}{\left(W_{b}\right)}_{jk}\\ \varpi =w+{\left[\mathrm{tr}\left(V_{b}\right)I-V_{b}\right]}^{-1}z\\ \Omega_{ij}=e_{ikj}\varpi_{k} \end{array}\right.,$$

where $e_{ikj}$ is the permutation symbol, and the left stretch tensor is

(55) $$V_{b}^{t}=V_{b}^{t-\Delta t}+\Delta t{\dot{V}}_{b}^{\Delta t},$$

in which ${\dot{V}}_{b}^{\Delta t}=L_{b}V_{b}^{t}-V_{b}^{t}\Omega$ is the left stretch tensor rate.

As of now, we have calculated the bond-associated unrotated deformation rate of the tensor $d_{b}$. In the JH2 constitutive relationship, the bond-associated unrotated Cauchy stress $\tau_{b}$ is calculated using the bond-associated unrotated deformation rate of the tensor $d_{b}$. At each time step, we will compute and store the rotated Cauchy stress $\sigma_{b}^{t-\Delta t}$ at the previous step through the stored unrotated Cauchy stress $\tau_{b}^{t-\Delta t}$, as

(56) $$\tau_{b}^{t-\Delta t}={\left(R_{b}^{t-\Delta t}\right)}^{T}\sigma_{b}^{t-\Delta t}R_{b}^{t-\Delta t},$$

where $V_{b}=I$ and $R_{b}=I$ when t=0.

We first assume that the material deformation is in the elastic stage, then the total elastic strain increment Δe and the partial elastic strain increment $\Delta e^{\mathrm{dev}}$ are respectively expressed as

(57) $$\begin{array}{cc} \Delta e=d_{b}\Delta t, & \Delta e^{\mathrm{dev}}=\Delta e-\frac{1}{3}I \end{array}.$$

At the current time t, the bond-associated unrotated trial Cauchy stress is

(58) $$\tau_{b}^{t,\mathrm{trial}}=\tau_{b}^{t-\Delta t}+\lambda \cdot \frac{1}{3}\mathrm{tr}\left(\Delta e\right)I+2Ge^{\mathrm{dev}}$$

where λ and G are the Lamé constants of solids. Based on Von–Mises plasticity theory, the bond-associated trial deviatoric stress tensor can be calculated as

(59) $$S_{b}=\tau_{b}^{t,\mathrm{trial}}-\frac{1}{3}\mathrm{tr}\left(\tau_{b}^{t,\mathrm{trial}}\right)I$$

The bond-associated equivalent Von–Mises yield stress is

(60) $$S_{b}^{\mathrm{VM}}=\sqrt{\frac{3}{2}{\left(S_{b}\right)}_{ij}{\left(S_{b}\right)}_{ij}}$$

The normalized strain rate is ${\dot{\varepsilon }}^{*}=d_{b}^{\mathrm{VM}}/{\dot{\varepsilon }}_{0}$, in which $d_{b}^{\mathrm{VM}}=\sqrt{\frac{2}{3}{\left(d_{b}\right)}_{ij}{\left(d_{b}\right)}_{ij}}$ is the total equivalent strain rate. Furthermore, the bond-associated hydrostatic pressure is calculated as

(61) $$P_{b}^{t}=\left\{\begin{array}{cc} K_{1}\mu_{b}^{t}+K_{2}{\left(\mu_{b}^{t}\right)}^{2}+K_{3}{\left(\mu_{b}^{t}\right)}^{3}+\Delta P_{b}^{t-\Delta t}, & \mathrm{If}\;\mu_{b}^{t}\geq 0\\ K_{1}\left(\mu_{b}^{t}\right)+\Delta P_{b}^{t-\Delta t}, & \mathrm{otherwise} \end{array}\right.$$

Based on Equation (60), the bond-associated yield function is expressed as

(62) $$f\left(\sigma_{b},{\dot{\varepsilon }}_{b}\right)=S_{{}_{b}}^{\mathrm{VM}}-\sigma_{b}^{*}\cdot \sigma_{\mathrm{HEL}}.$$

If $f\left(\sigma_{b},{\dot{\varepsilon }}_{b}\right)<0$, the material is in the elastic stage, and the bond-associated unrotated Cauchy stress is equal to the bond-associated unrotated trail Cauchy stress. Otherwise, the material yields, and we need to recalculate the bond-associated true elastic strain increment, plastic strain increment, and unrotated Cauchy stress.

For the three-dimensional stress state, the bond-associated plastic strain rate vector is calculated according to Prandtl–Reuss flow law as

(63) $${\dot{\varepsilon }}_{b}^{p}=\dot{\lambda }\frac{\partial f\left(\sigma_{b},{\dot{\varepsilon }}_{b}\right)}{\partial S_{b}}=\dot{\lambda }a,$$

in which $\dot{\lambda }$ is the bond-associated plastic strain rate multiplier, and a is an orthogonal vector. When the plastic deformation occurs, the stress rate or stress increment is perpendicular to the yield surface, as follows:

(64) $$f\left(\sigma_{b},{\dot{\varepsilon }}_{b}\right)=a^{T}{\dot{\sigma }}_{b}.$$

When the material yields, the total strain rate is decomposed into the elastic component and the plastic component. The relationship between stress and stress rate can be expressed as

(65) $${\dot{\sigma }}_{b}=C\left({\dot{\varepsilon }}_{b}-{\dot{\varepsilon }}_{b}^{p}\right)=C\left({\dot{\varepsilon }}_{b}-\dot{\lambda }a\right),$$

where C is a stiffness matrix of the material. The bond-associated plastic strain rate multiplier $\dot{\lambda }$ is

(66) $$\dot{\lambda }=\frac{a^{T}C{\dot{\varepsilon }}_{b}}{a^{T}Ca}.$$

By considering Equations (64)–(66), the bond-associated equivalent plastic strain increment is

(67) $$\Delta \varepsilon_{b}^{p}=\frac{a^{T}C{\dot{\varepsilon }}_{b}\Delta T}{\sqrt{2}a^{T}Ca}\sqrt{{\left(S_{b}^{xx}-S_{b}^{yy}\right)}^{2}+{\left(S_{b}^{xx}-S_{b}^{zz}\right)}^{2}+{\left(S_{b}^{yy}-S_{b}^{zz}\right)}^{2}+6\left[{\left(S_{b}^{xy}\right)}^{2}+{\left(S_{b}^{yz}\right)}^{2}+{\left(S_{b}^{zx}\right)}^{2}\right]}.$$

If the bond-associated equivalent plastic strain increment is not currently zero, the bond-associated fracture damage factor accumulates as

(68) $$D_{b}^{t}=D_{b}^{t-\Delta t}+\frac{\Delta \varepsilon_{b}^{p}}{\varepsilon_{b,f}^{p}}$$

The incremental energy loss is

(69) $$\Delta U=U^{t-\Delta t}-\frac{{\left(\sigma_{\mathrm{HEL}}\cdot \sigma_{b}^{*}\right)}^{2}}{6G}.$$

In the current configuration, the bond-associated final hydrostatic pressure is recalculated as

(70) $$P_{b}^{t}=\frac{1}{3}\mathrm{tr}\left(\tau_{b}^{t,\mathrm{trail}}\right)+\Delta P_{b}^{t}.$$

Finally, the bond-associated unrotated Cauchy stress tensor and rotated Cauchy stress at the current time step can be updated to

(71) $$\tau_{b}^{t}=P_{b}^{t}I+S_{b},$$

(72) $$\sigma_{b}^{t}=R_{b}^{t}\tau_{b}^{t}{\left(R_{b}^{t}\right)}^{T}.$$

Thereafter, we can calculate the stress tensor $P_{b}$ in Equation (13), and then update the force state $\underline{T_{b}}$ at the current time step in Equation (12).

### 2.4. Other Zero-Energy Mode Control Schemes

In addition to the bond-associated deformation gradient scheme proposed in BA-NOSB PD, different zero-energy mode control schemes have been proposed to suppress or eliminate numerical oscillations. In this section, we briefly introduce four zero-energy mode control schemes based on the supplemental force state. In the NOSB model based on the supplemental force state, the total force state can be written as

(73) $${\underline{T}}^{\mathrm{total}}\left(X,t\right)\langle \xi \rangle =\underline{T}\left(X,t\right)\langle \xi \rangle +{\underline{T}}^{s}\left(X,t\right)\langle \xi \rangle ,$$

in which the original force state $\underline{T}$ is defined in Equation (5), and ${\underline{T}}^{s}$ is the corresponding supplemental force state.

- Control Scheme I:

In Littlewood’s work [26], a penalty scheme is used to control the zero-energy mode. This is similar to the method of controlling deformable hourglass modes in finite element analysis by adding the hourglass force to the original force state. The added hourglass force is expressed as

(74) $${\underline{T}}^{s}\left(X,t\right)\langle \xi \rangle =-c_{\mathrm{hg}}c\frac{h_{\mathrm{proj}}}{\left\|\xi \right\|}\frac{\xi +\eta }{\left\|\xi +\eta \right\|}\Delta V_{X}\Delta V_{X^{\prime }},$$

in which $c_{\mathrm{hg}}$ is a user parameter controlling the level of the hourglass force, c is the bond constant of the PMB model, and $c=18K/\pi \delta^{4}$.

The projection of the hourglass vector h on the relative position vector ξ+η under the current configuration can be expressed as

(75) $$h_{\mathrm{proj}}=h\cdot \left(\xi +\eta \right),$$

where the hourglass vector h is

(76) $$h={y^{\prime }}^{*}-y^{\prime },$$

in which the position of the neighbor $X^{\prime }$ in the current configuration is calculated by the approximative deformation gradient F as:

(77) $${y^{\prime }}^{*}=y+F\xi .$$

- Control Scheme II:

In addition, Silling [27] derived the stability conditions of the NOSB PD model and proposed a new supplemental force state that can be written as

(78) $${\underline{T}}^{s}\left(X,t\right)\langle \xi \rangle =\underline{\omega }\langle \left\|\xi \right\|\rangle \frac{Gc_{1}}{\omega_{0}}\underline{z}\langle \xi \rangle ,$$

where G is a user parameter controlling the level of the supplemental force state, and $c_{1}=c/\delta$ is the micro-modulus in BB PD. The sum of the influence function values of all bonds at the material point X is

(79) $$\omega_{0}=\int_{H_{X}}\underline{\omega }\langle \left\|\xi \right\|\rangle dV_{X^{\prime }}.$$

Furthermore, the non-uniform deformation state can be expressed as

(80) $$\underline{z}\langle \xi \rangle =\underline{Y}\langle \xi \rangle -F\xi .$$

- Control Scheme III:

Li et al. [28] proposed a novel supplemental force state characterizing the non-uniform deformation state based on the linearized BB PD theory, which can be written as

(81) $${\underline{T}}^{s}\left(X,t\right)\langle \xi \rangle =\underline{\omega }\langle \left\|\xi \right\|\rangle \underline{C}\langle \xi \rangle \underline{z}\langle \xi \rangle ,$$

where $\underline{C}\langle \xi \rangle$ is the elasticity coefficient tensor, defined as

(82) $$\underline{C}\langle \xi \rangle =c\xi ⊗\xi /{\left\|\xi \right\|}^{3}.$$

Compared with the control schemes I and II, this scheme does not require the user parameter, and avoids the complicated parameter adjustment process.

- Control Scheme IV:

Wan et al. [29] proposed a new supplemental force state directly within the NOSB PD model, which can be expressed as

(83) $${\underline{T}}^{s}\left(X,t\right)\langle \xi \rangle =\underline{\omega }\langle \left\|\xi \right\|\rangle C_{e}K^{-1}\underline{z}\langle \xi \rangle ,$$

where $C_{e}$ is the elastic modulus tensor. Similar to the Control Scheme III, this supplemental force state also does not contain the user parameter.

### 2.5. Numerical Discretization and Implementation

#### 2.5.1. Numerical Discretization

The solution to the PD governing equation of motion usually requires discrete spatial domain and time domain for numerical integration. In the PD theory, the geometric model of the problem domain is presented by discretized material points. Accordingly, in the BA-NOSB PD model, the discrete form of the equation of motion for the material point $X_{i}$ at the instant of t can be written as

(84) $$\rho_{0}(X_{i})\ddot{u}\left(X_{i},t\right)=\sum_{j}^{N}\left[\underline{T_{b}}\left(X_{i},t\right)\langle \xi_{ij}\rangle -\underline{T_{b}}\left(X_{j},t\right)\langle \xi_{ji}\rangle \right]V_{X_{j}}+b\left(X_{i},t\right),$$

where N is the number of neighbors for the material point $X_{i}$, and $\xi_{ij}=X_{j}-X_{i}$.

The discrete forms of the bond-associated shape tensor and deformation gradient tensor about the bond $\xi_{ij}$ at the material point $X_{i}$ is

(85) $$K_{b}(X_{i},\xi_{ij})=\sum_{k=1}^{N_{b}}\underline{\omega }\langle \left\|\xi_{ik}\right\|\rangle \xi_{ik}⊗\xi_{ik}\Delta V_{X_{k}},$$

(86) $$F_{b}(X_{i},\xi_{ij})=\left[\sum_{k=1}^{N_{b}}\underline{\omega }\langle \left\|\xi_{ik}\right\|\rangle \left(\xi_{ik}+\eta_{ik}\right)⊗\xi_{ik}\Delta V_{X_{k}}\right]K_{b}^{-1}(X_{i},\xi_{ij}),$$

where $N_{b}$ is the number of bond-associated neighbors with respect to the bond $\xi_{ij}$ at the material point $X_{i}$.

Finally, we use the explicit Velocity–Verlet integration algorithm to implement the time-integral scheme, which can be expressed as

(87) $${\dot{u}}_{t+\Delta t}={\dot{u}}_{t}+\frac{1}{2}\left({\ddot{u}}_{t}+{\ddot{u}}_{t+\Delta t}\right)\Delta t,$$

(88) $$u_{t+\Delta t}=u_{t}+{\dot{u}}_{t}\Delta t+\frac{1}{2}{\ddot{u}}_{t}{\left(\Delta t\right)}^{2},$$

where Δt is the time step size of the time-integral cycle.

#### 2.5.2. Artificial Viscosity

In numerical simulations of the impact or penetration problems, jump oscillations often occur in the impact domain, which may lead to unphysical deformation modes. Artificial viscosity [50,51] is widely used in penetration or impact simulations to improve the stability of numerical algorithms and to prevent unphysical overlapping or interpenetration between particles. In this work, the artificial viscosity implemented in PD theory by Lai et al. [20] is used for impact problems, which can be expressed as

(89) $$\prod_{ij}=\left\{\begin{array}{cc} \frac{-\alpha_{\prod }{\bar{c}}_{ij}\phi_{ij}+\beta_{\prod }\phi_{ij}{}^{2}}{{\bar{\rho }}_{ij}}, & v_{ij}\cdot x_{ij}<0\\ 0, & v_{ij}\cdot x_{ij}\geq 0 \end{array}\right.,$$

where

(90) $$\phi_{ij}=\frac{\delta_{ij}v_{ij}\cdot x_{ij}}{{\left\|x_{ij}\right\|}^{2}+\varphi^{2}},$$

(91) $${\bar{c}}_{ij}=\frac{1}{2}\left(c_{i}+c_{j}\right),$$

(92) $${\bar{\rho }}_{ij}=\frac{1}{2}\left(\rho_{i}+\rho_{j}\right),$$

(93) $$\delta_{ij}=\frac{1}{2}\left(\delta_{i}+\delta_{j}\right),$$

(94) $$v_{ij}=v_{i}-v_{j}\;\mathrm{and}\;x_{ij}=x_{i}-x_{j}.$$

In the above Equations, $\alpha_{\prod }$ and $\beta_{\prod }$ are constants with values of about 1.0. The factor $\varphi =0.1\delta_{ij}$ is taken into account to avoid numerical divergence. The velocity vector of the particle is v. The sound speed, mass density, and horizon of the particle are c, ρ, and δ, respectively.

The artificial viscosity state introduced can be written as

(95) $$\underline{\prod }\left(X_{i},t\right)\langle \xi_{ij}\rangle =\omega \langle \left\|\xi_{ij}\right\|\rangle \nabla_{i}\prod_{ij}\langle \xi_{ij}\rangle ,$$

in which ∇ is the vector differential operator. When $v_{ij}\cdot x_{ij}<0$,

(96) $$\nabla_{i}\prod_{ij}=\frac{-\alpha_{\prod }{\bar{c}}_{ij}+2\phi_{ij}\beta_{\prod }}{{\bar{\rho }}_{ij}}\frac{\partial \phi_{ij}}{\partial x_{i}},$$

where

(97) $$\begin{array}{c} \frac{\partial \phi_{ij}}{\partial x_{i}}=\delta_{ij}\left(-\frac{\partial v_{i}}{\partial x_{i}}\underline{Y}\langle \xi_{ij}\rangle -\dot{\underline{Y}}\langle \xi_{ij}\rangle \right)\frac{1}{{\left\|\underline{Y}\langle \xi_{ij}\rangle \right\|}^{2}+\varphi^{2}}\\ +\delta_{ij}\left(\dot{\underline{Y}}\langle \xi_{ij}\rangle \cdot \underline{Y}\langle \xi_{ij}\rangle \right)\frac{2\underline{Y}\langle \xi_{ij}\rangle }{{\left({\left\|\underline{Y}\langle \xi_{ij}\rangle \right\|}^{2}+\varphi^{2}\right)}^{2}} \end{array},$$

where

(98) $$\frac{\partial v_{i}}{\partial x_{i}}=\left(\sum_{j=1}^{N}\underline{\omega }\langle \left\|\xi_{ij}\right\|\rangle \dot{\underline{Y}}\langle \xi_{ij}\rangle ⊗\xi_{ij}\Delta V_{X_{j}}\right)K_{X_{i}}^{-1}F_{X_{i}}^{-1}$$

(99) $$\dot{\underline{Y}}\langle \xi_{ij}\rangle =v\left(X_{j},t\right)-v\left(X_{i},t\right)$$

It is worth noting that the introduced artificial viscosity is not physical viscosity, but only numerical correction. This correction prevents the unphysical deformation behavior of particles in dynamic fracture problems such as impact or penetration.

#### 2.5.3. Contact Algorithm

**Figure 4:**
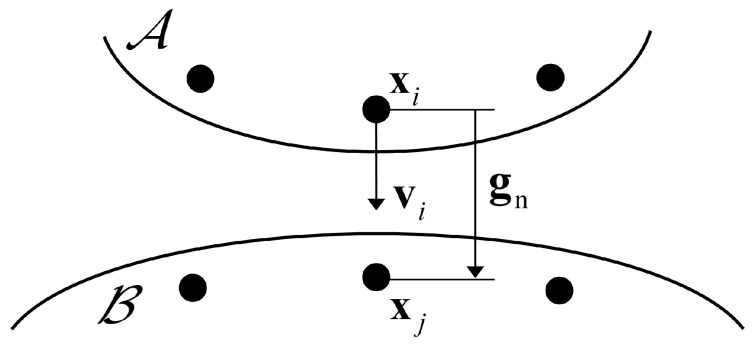
Schematic diagram of the contact surfaces under normal impact [20].

The impact or penetration simulation is a critical problem often encountered in fields such as industrial manufacturing, military equipment, and scientific research. Such problems often involve contact and collision analysis between two or more objects. Among the interface contact algorithms [52,53,54] that have been developed and used, the penalty method is the most commonly used [55]. In this work, the penalty method implemented in PD theory by Lai et al. [20] will be used to model impact problems. Figure 4 shows the normal impact problem for two objects A and B. The material points $X_{i}$ and $X_{j}$ are on the surfaces of the objects A and B, respectively. At the initial moment, the material point $X_{j}$ remains stationary, and the velocity of the material point $X_{i}$ is $v_{i}$. The relative position vector at the current time step is $g_{n}=x_{j}-x_{i}$, and $l=\left\|g_{n}\right\|$.

When $l<l_{c}$, contacts occur between the material points of the master and slave, and a contact bond $\xi_{ij}=X_{j}-X_{i}$ is formed. At the current moment t, the contact force of the material points $X_{j}$ on $X_{i}$ can be expressed as

(100) $$f_{c}\left(X_{i},t\right)\langle \xi_{ij}\rangle =\left\{\begin{array}{cc} -g_{n}/l\cdot C_{\xi }V_{X_{i}}\ln\left(l/l_{c}\right), & \mathrm{if}\;l<l_{c}\\ 0, & \mathrm{otherwise} \end{array}\right.,$$

where $l_{c}$ is a standard value and is usually chosen to be slightly smaller than the grid size of the discretization. The contact stiffness $C_{\xi }$ is defined on the contact bond, and can be calculated as

(101) $$C_{\xi }=\min\left(\rho_{i},\rho_{j}\right)\times 2\times 10^{n},$$

where $\rho_{i}$ and $\rho_{j}$ are the mass densities, and n is the penalty factor. It is worth noting that contact forces can only be repulsive forces.

Therefore, the total contact force at the material point $X_{i}$ is expressed as

(102) $$F_{c}\left(X_{i},t\right)=\begin{array}{cc} \sum_{i=1}^{M}f_{c}\left(X_{i},t\right)\langle \xi_{ij}\rangle , & \xi_{ij}<\delta_{c} \end{array},$$

where M is the number of material points forming contact bonds, and $\delta_{c}$ is a contact horizon.

## 3. Numerical Examples

In this section, four numerical examples are established to verify and demonstrate the modified BA-NOSB PD model. In the first part, two numerical examples are used to numerically model the uniaxial tension experiments of elastic bars under different loading forms to verify the effectiveness and robustness of the modified model. In the second part, two numerical experiments of the edge-on impact and normal impact of ceramics are carried out to demonstrate the ability of the modified model for impact problems of quasi-brittle materials. Good agreement is shown by comparing PD simulation results with references.

### 3.1. Uniaxial Tension of an Elastic Bar under Stress Loading

**Figure 5:**
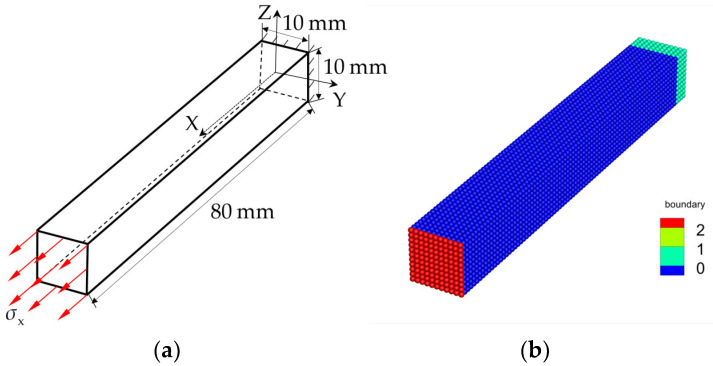
Schematic diagram of uniaxial tension for an elastic bar under stress loading. (**a**) Computational model. (**b**) Discretization.

In this section, the numerical modeling of the uniaxial tension test of a three-dimensional elastic bar under stress loading is carried out, and the computational model is seen in Figure 5a. The configuration of the model is $80\times 10\times 1{0\;\mathrm{mm}}^{3}$. A fixed constraint is applied at one end of the bar, and a dynamic tensile stress $\sigma_{x}=5.0\;\mathrm{kPa}$ is applied to the other end. The specimen material used in this work is an Al_2_O_3_ ceramic with elastic modulus E=220 GPa. The parameters of the JH2 constitutive model for the Al_2_O_3_ ceramic are listed in Table 1. As shown in Figure 5b, the loading end consists of one layer of red material points, and the fixed end consists of m layers of light green material points. In the PD simulation, the boundary conditions are as follows: the dynamic tensile stress is applied to red material points, and the fixed constraint is applied to light green material points, as seen in Figure 5b. The ceramic bar is discretized into cubic particles with the grid size Δx, where $\Delta x=1.0\times 10^{-3}\;m$. The horizon size of the discretization model is δ=m·Δx, and the time step size is $\Delta t=1.0\times 10^{-8}\;s$. The model is discretized into 9801 material points.

This section will verify the effectiveness of controlling zero-energy modes of the modified BA-NOSB PD model and compare the numerical stability of different control schemes. The axial displacement simulation results from different control schemes are compared with those of the original NOSB PD model. In comparison and analysis, simulation results at t=100 μs are selected. In this example, the grid size Δx of the discretization model remains fixed, and five cases with horizon factors m of 1.5, 2.0, 3.0, 4.0, and 5.0 are selected for comparison and analysis of the results.

**Figure 6:**
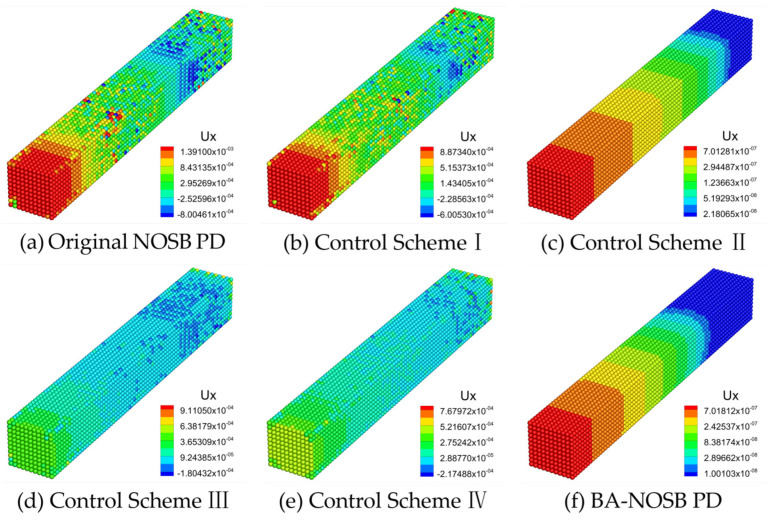
Comparison of axial displacement contours with m=1.5.

Figure 6 shows the axial displacement contours of the 3D ceramic bar calculated by the original NOSB PD model, four other zero-energy mode control schemes, and the modified BA-NOSB PD model when m=1.5. The displacement solution obtained from the original NOSB PD model in Figure 6a shows very violent oscillations. The result based on the Control Scheme I in Figure 6b is similar to that of the original NOSB PD model, and there are also severe oscillations. As shown in Figure 6d,e, the displacement solutions obtained by the Control Schemes III and IV are obviously incorrect, and there are obvious oscillations near the fixed end. The displacement solutions of the Control Scheme II and the modified BA-NOSB PD model show discontinuity near the fixed end, and the results of the Control Scheme II are smoother and more continuous than that of the modified BA-NOSB PD model, as shown in Figure 6c,f.

**Figure 7:**
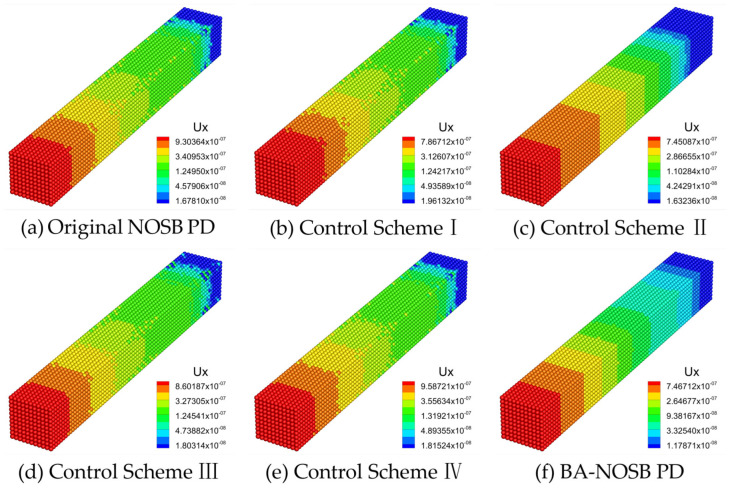
Comparison of axial displacement contours with m=2.0.

Figure 7 shows the axial displacement contours calculated by the different PD models when m=2.0. The displacement solution obtained from the original NOSB PD model in Figure 7a shows slight oscillations, which are more significant near the fixed end, and the displacement contour in the middle of the bar is not smooth and continuous. The results of the Control Schemes I, III, and IV are similar to that of the original NOSB PD model, all with slight oscillations, as seen in Figure 7b,d,e. In addition, the Control Scheme II and the modified BA-NOSB PD model both obtain smooth displacement solutions, but the results of the Control Scheme II have slight oscillations near the fixed end, as seen in Figure 7c,f.

**Figure 8:**
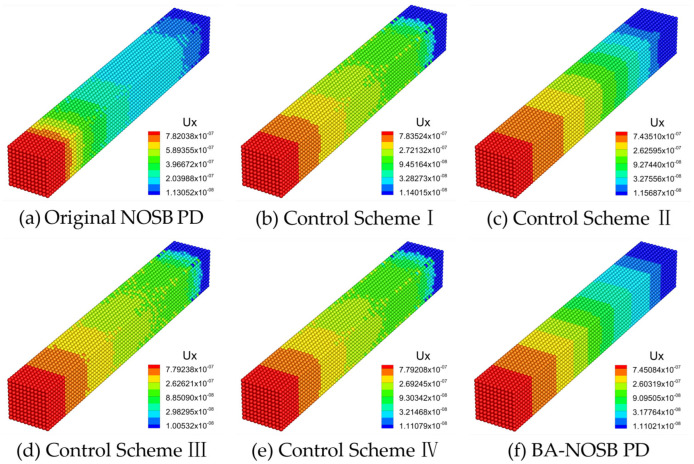
Comparison of axial displacement contours with m=3.0.

Figure 8 shows the axial displacement contours calculated by the different PD models when m=3.0. The displacement solution obtained from the original NOSB PD model in Figure 8a shows obvious oscillations near the fixed end and slight oscillations near the loading end. In addition, the displacement contour in the middle of the bar is not smooth and continuous. The displacement solutions obtained by the Control Schemes I, III, and IV are similar. These results have obvious oscillations near the fixed end, and the displacement contours in the middle of the bar are not smooth and continuous, as shown in Figure 8b,d,e. The result obtained by the Control Scheme II in Figure 8c has slight oscillations near the fixed end. In addition, the modified BA-NOSB PD model provides a smooth displacement solution that can effectively eliminate numerical oscillations, as shown in Figure 8f.

**Figure 9:**
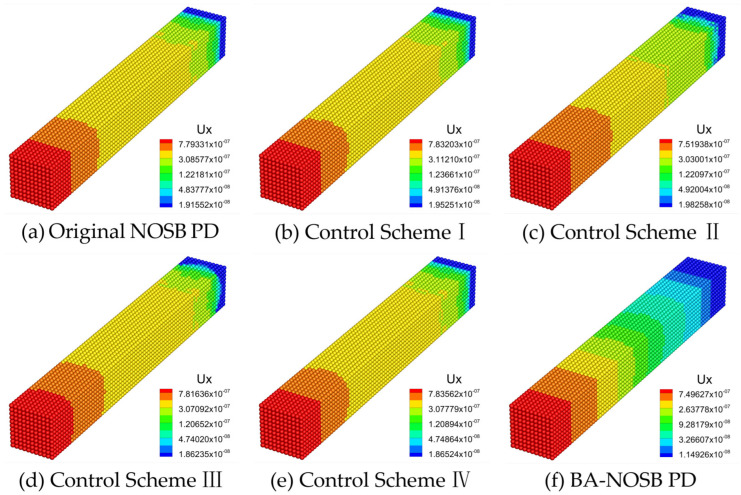
Comparison of axial displacement contours with m=4.0.

Figure 9 shows the axial displacement contours calculated by the different PD models when m=4.0. There are slight oscillations in the displacement solution obtained from the original NOSB PD model in Figure 9a, and the displacement contour in the middle of the bar is not smooth and continuous. The results of the Control Schemes I, III, and IV are similar. Moreover, these results have slight oscillations near the fixed end, and the displacement contours in the middle of the bar are not smooth and continuous, as shown in Figure 9b,d,e. The result obtained by the Control Scheme II in Figure 9c shows slight oscillations near the fixed end. The modified BA-NOSB PD model provides smooth and continuous displacement contours, as shown in Figure 9f.

**Figure 10:**
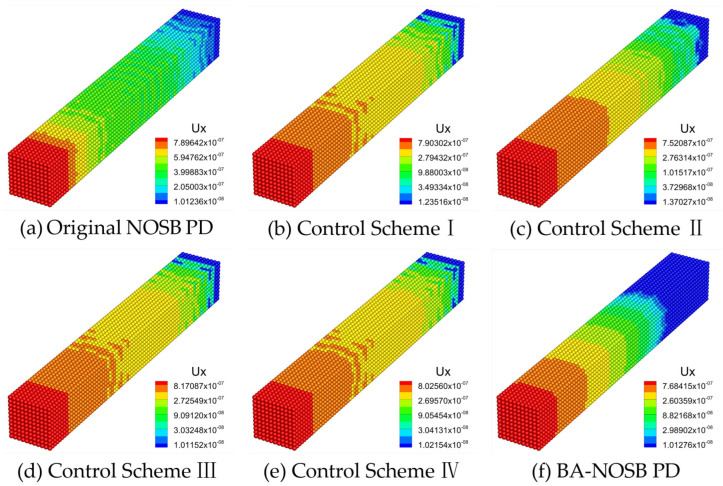
Comparison of axial displacement contours with m=5.0.

Figure 10 shows the axial displacement contours calculated by the different PD models when m=5.0. The displacement solution obtained from the original NOSB PD model in Figure 10a shows obvious oscillations near both the fixed end and the loading end. The results of the Control Schemes I, III, and IV all show obvious oscillations at the fixed end and in the middle of the bar, as shown in Figure 10b,d,e. In addition, the result of the Control Scheme II has slight oscillations near the fixed end, and the displacement contour in the middle of the bar is not smooth and continuous, as shown in Figure 10c. The displacement solution obtained from the modified BA-NOSB PD model in Figure 10f has no obvious oscillations, but it does show discontinuous features near the fixed end.

This section simulates a uniaxial tension experiment of a 3D ceramic bar under stress loading, and compares the effectiveness of different zero-energy mode control schemes to suppress or eliminate numerical oscillations. The following conclusions can be drawn from the above comparison and analysis. First, the simulation results of the original NOSB PD model show different degrees of oscillations for different values of m. Similarly, the results of the Control Schemes I, III, and IV all show different degrees of oscillations for different values of m, which fail effectively to suppress or eliminate numerical oscillations. In addition, the Control Scheme II effectively eliminates oscillations at smaller values of m (1.5≤m≤3.0), while slight oscillations exist at larger values of m (4.0≤m≤5.0). Finally, the modified BA-NOSB PD model can effectively eliminate oscillations for different values of m, showing slight discontinuous characteristics in the case of m=1.5 or m=5.0.

On the other hand, by analyzing the above simulation results with numerical oscillations, two conclusions can be drawn. First, the oscillations of the result are more obvious when a large or small horizon factor m (m≤2.0 or m≥4.0) is selected, while the oscillations of the result are lighter when a moderate horizon factor m (m=3.0) is selected. Secondly, the oscillations of the simulation results mainly exist in the region near the boundaries, such as the fixed end or the loading end.

In summary, the modified BA-NOSB PD model can effectively eliminate the numerical oscillations and unphysical deformation modes. Compared to four other zero-energy mode control schemes, the modified model has a more robust zero-energy mode control capability.

### 3.2. Uniaxial Tension of an Elastic Bar under Velocity Loading

In order to more accurately implement the JH2 constitutive model in the framework of BA-NOSB PD theory, the bond-associated volumetric strain $\mu_{b}$ is defined in Section 2.2.1. In addition, for the SB PD theory, a new treatment strategy for broken bonds is proposed in Section 2.2.3. In this section, for the volumetric strain in the equation of state and the broken bonds, the following three solutions are presented to modify the original BA-NOSB PD model, as shown in Table 2.

**Figure 11:**
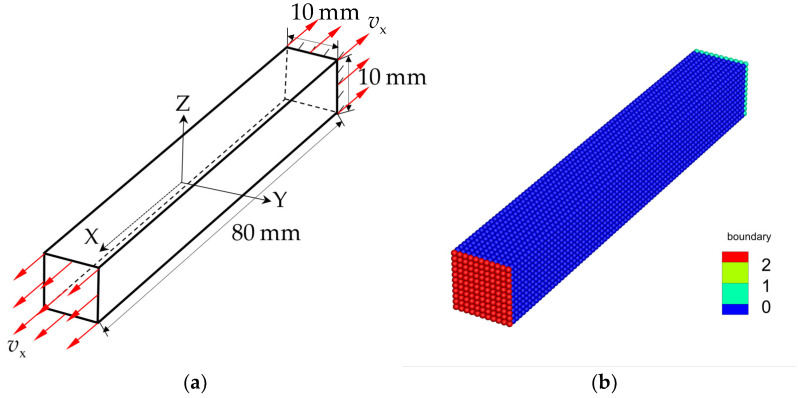
Schematic diagram of uniaxial tension of an elastic bar under velocity loading. (**a**) Computational model. (**b**) Discretization.

To evaluate the above three solutions, the uniaxial tension experiment of the 3D elastic bar under velocity loading will be numerically modeled in this section, and the computational model is shown in Figure 11a. The configuration of the model is $80\times 10\times 10{\;\mathrm{mm}}^{3}$. At the initial moment, an instantaneous tensile velocity load of the same magnitude is exerted on both ends of the bar, and the magnitude of the velocity load is $v_{X}=0.1\;m/s$. The sample material is the Al_2_O_3_ ceramic with the same material parameters as in Section 3.1. The JH2 constitutive model parameters of Al_2_O_3_ ceramic are shown in Table 1. In the PD simulation, the tensile velocity loads are applied to red and light green material points, respectively, as seen in Figure 11b. The grid size of the discretization is $\Delta x=1.0\times 10^{-3}\;m$, and the horizon factor is chosen as m=3.0. The time step size is $\Delta t=1.0\times 10^{-8}\;s$. The model is discretized into 9801 material points.

**Figure 12:**
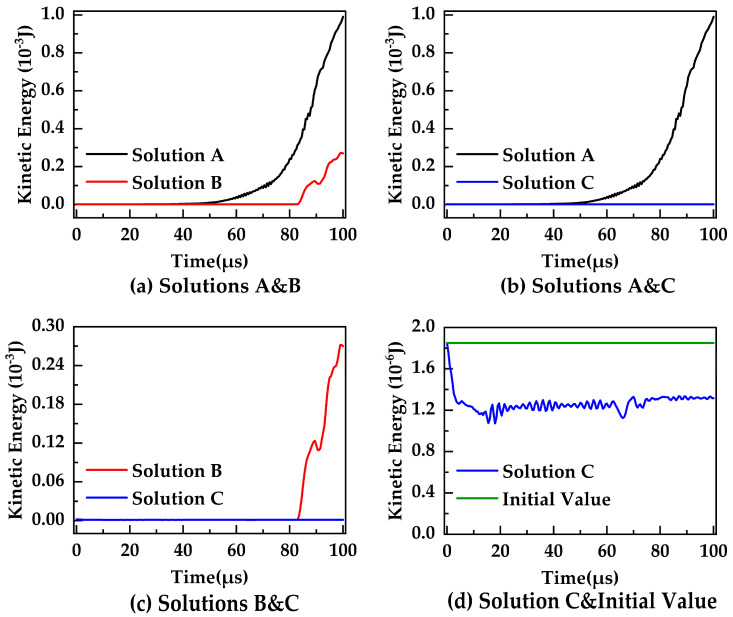
Convergence curves for the system kinetic energy with different solutions.

From the perspective of kinetic energy convergence, the change in the system kinetic energy over time will be compared to discuss and evaluate the above three solutions. At the initial moment, both ends of the bar are subjected to a tensile velocity load, and the initial system kinetic energy is $1.85\times 10^{-6}\;J$. This initial kinetic energy is also the total energy of the whole system. Figure 12 shows the change in the total kinetic energy of the PD simulation system based on the three solutions over time. For Solution A, the ceramic bar begins to show damage at t=65 μs, and the system kinetic energy increases sharply to $9.902\times 10^{-4}\;J$ from the damage occurrence to complete brittle fracture. For Solution B, the bar begins to show damage at t=82.5 μs, and the system kinetic energy increases significantly to $2.701\times 10^{-4}\;J$ from the damage occurrence to complete brittle fracture. The final system kinetic energy for Solutions A and B is much higher than the initial value, and the system kinetic energy will increase significantly after the damage occurs, as shown in Figure 12a. Completely different from Solutions A and B, the system kinetic energy of Solution C does not change significantly from the damage appearance to brittle fracture, as shown in Figure 12b,c. Further, as shown in Figure 12c, the system kinetic energy of Solution B increases rapidly after the damage occurs, while the system kinetic energy of Solution C remains stable. Compared to the initial value, it can be seen that the system kinetic energy of Solution C is always lower than the initial value and does not increase significantly or far exceed the initial value, as shown in Figure 12d.

By comparing and analyzing the convergence curves of the system kinetic energy with the three solutions, the results are summarized as follows. First, the system kinetic energy of Solutions A and B both increase dramatically after the damage occurs, and the final values are much larger than the initial value. Second, the system kinetic energy of Solution C changes steadily over time and is always below the initial value. The results show that only Solution C satisfies the requirement of kinetic energy convergence, while both Solutions A and B do not. In addition, the system kinetic energy of Solutions A and B in the final stage is much greater than the total energy, which means that neither meets the requirement of energy conservation. From the perspective of kinetic energy convergence, Solution C provides better accuracy and robustness for the BA-NOSB PD model and is chosen as the final modified solution.

### 3.3. Edge-On Impact Simulation of Ceramics

**Figure 13:**
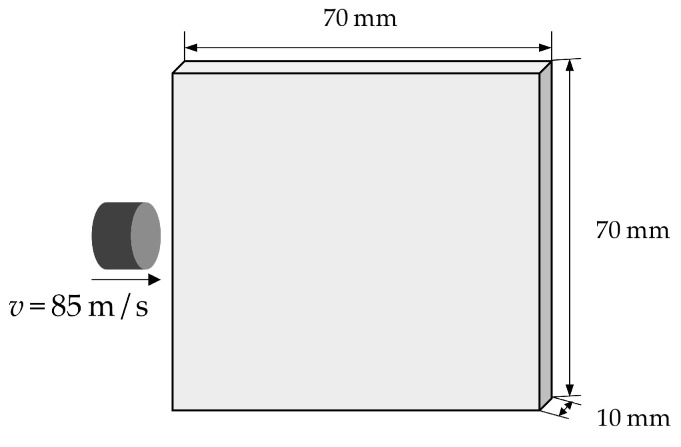
Computational model of ceramics under edge-on impact.

In this section, the numerical experiments on the edge-on impact of ceramic plates will be carried out. The contact algorithm in Section 2.5.3 will be used to describe the interaction between the cylindrical projectile and the target plate. Further, the artificial viscosity introduced in Section 2.5.2 will be used to prevent unphysical oscillations or interpenetration in the impact zone. Figure 13 shows the computational model of a ceramic plate under edge-on impact. The configuration of the target plate is $70\times 70\times 10{\;\mathrm{mm}}^{3}$. In addition, the projectile is a cylinder with a diameter of 16 mm and a height of 21 mm. The target plate is the Al_2_O_3_ ceramic, and its material parameters and JH2 constitutive model parameters are the same as those in Section 3.1. For simplicity, the projectile is regarded as a rigid body with density $\rho =8060{\;\mathrm{kg}/m}^{3}$. With the same set-up as in the reference, free boundary conditions are applied to the ceramic target plate, and the impact velocity of the projectile is v=85 m/s. In the PD simulation, the grid size of the discretization is $\Delta x=1.0\times 10^{-3}\;m$, and the horizon factor is chosen as m=3.0. The time step size used in the edge-on impact is $\Delta t=2.0\times 10^{-9}\;s$. The computational model is discretized into 55,510 material points.

**Figure 14:**
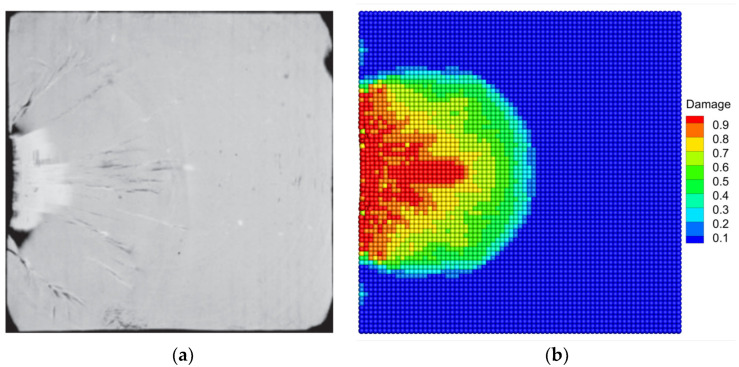
Comparison of fracture patterns for the Al_2_O_3_ ceramic plate under edge-on impact when t=9.7 μs (**a**) Experimental [57]; (**b**) PD simulation.

Figure 14 shows the fracture patterns of the Al_2_O_3_ ceramic plate under edge-on impact in the experiment and numerical simulation. Comparing the PD simulation result with the fracture image provided by Strassburger [57], good agreement can be found. Specifically, both the delta-like fracture pattern and the radial crack propagation paths in Figure 14a can be observed from the simulation result in Figure 14b. In addition, the simulation result shows the characteristics of the fragmentation of the target edge and the semi-elliptical damage zone, which is very similar to the fracture behavior observed in the experiment. The results demonstrate that the modified BA-NOSB PD model can accurately reproduce the fracture pattern and the crack propagation paths of the Al_2_O_3_ ceramic plate under the edge-on impact experiment.

**Figure 15:**
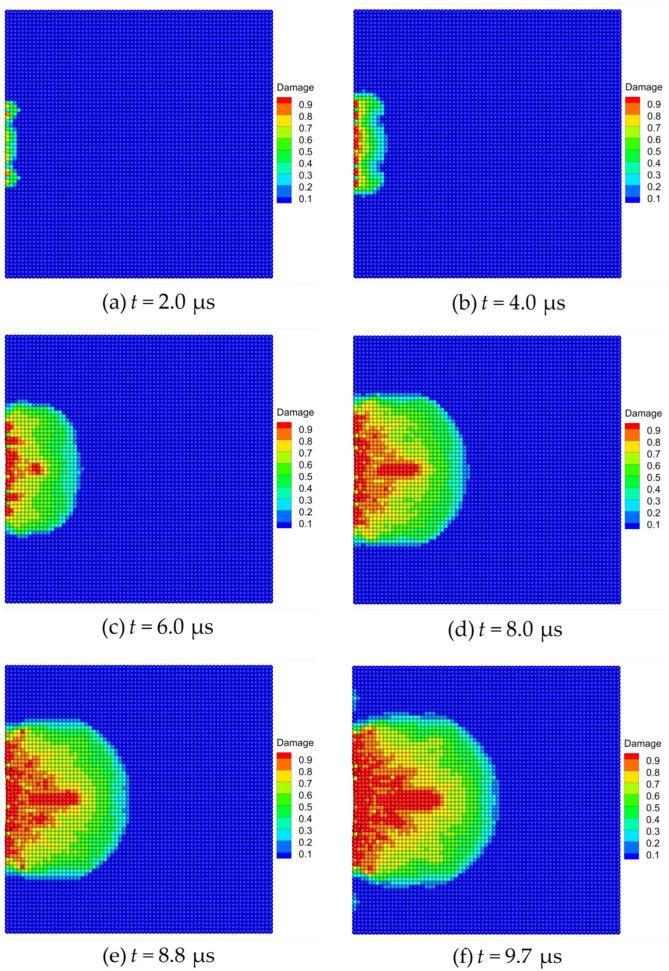
Damage evolution patterns of the Al_2_O_3_ ceramic plate under edge-on impact.

The damage evolution and crack propagation of the Al_2_O_3_ ceramic plate during edge-on impact are shown in Figure 15. After the cylindrical projectile hit the ceramic target plate, it began to penetrate the ceramic plate. At t=2.0 μs, the left edge of the target plate began to display gear-shaped damage, as shown in Figure 15a. At t=4.0 μs, the damage of the target plate further increases and extends along the impact direction, as shown in Figure 15b. With the continuous action of the projectile, five radial cracks form on the edge of the target plate, as shown at the instant of t=6.0 μs in Figure 15c. At t=8.0 μs, a triangular crack extension zone and a semicircular damage zone are formed on the target plate, as shown in Figure 15d. Subsequently, at t=8.8 μs, a corrugated damage zone and radial cracks are formed on the edge of the target plate, as shown in Figure 15e. Finally, with the weakening of the projectile action, the damage tended to be stable, and a fracture pattern similar to an alluvial delta is formed on the front surface of the ceramic plate, as shown at the instant of t=9.7 μs in Figure 15f.

### 3.4. Normal Impact Simulation of Ceramics

**Figure 16:**
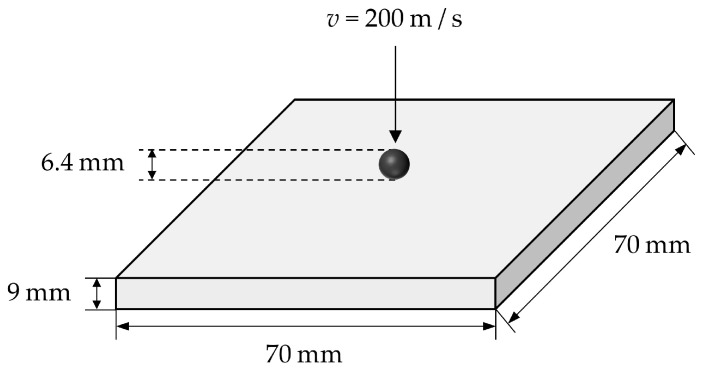
Computational model of ceramics under normal impact.

In this section, the normal impact experiment on a ceramic plate will be numerically modeled. The contact algorithm in Section 2.5.3 will be used to describe the interaction of the spherical projectile with the target plate. The artificial viscosity in Section 2.5.2 is incorporated to avoid unphysical oscillations or interpenetration in the impact area. The computational model of the normal impact simulation for the ceramic plate is shown in Figure 16. The configuration of the ceramic plate is $70\times 70\times 9{\;\mathrm{mm}}^{3}$. Moreover, the projectile is a sphere with a diameter of 6.4 mm. The material of the target plate is the Al_2_O_3_ ceramic with the same material parameters as in Section 3.1. The JH2 constitutive model parameters of the Al_2_O_3_ ceramic are shown in Table 1. For simplicity, the projectile material is set to be rigid, and its density is $\rho =7800{\;\mathrm{kg}/m}^{3}$. With the same set-up as in the reference, the ceramic plate is subjected to free boundary conditions, and the impact velocity of the projectile is v=200 m/s. In the PD simulation, the grid size of the discretization is $\Delta x=1.0\times 10^{-3}\;m$, and the horizon factor is chosen as m=3.0. The time step size in normal impact is $\Delta t=2.0\times 10^{-9}\;s$. The model is discretized into 47,256 material points.

**Figure 17:**
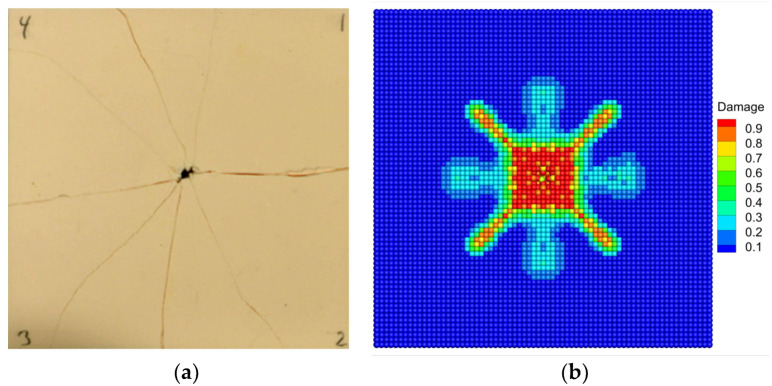
Comparison of fracture patterns for the Al_2_O_3_ ceramic plate under normal impact. (**a**) Experimental [58]; (**b**) PD simulation.

Figure 17 shows the fracture patterns of the upper surface of the Al_2_O_3_ ceramic plate under normal impact obtained from the experiment and PD simulation. Comparing the experimental result [58] and the simulation result, good agreement can be found on the fracture patterns. The PD simulation result in Figure 17b accurately reproduces the eight radial cracks along the 0°, 45°, 90°, 135°, 180°, 225°, 270°, and 315° directions of the ceramic plate in the experiment. In addition, the PD simulation result can accurately reproduce the characteristics of fragmentation and collapse in the center of the plate caused by projectile penetration. These fracture characteristics agree well with the experimental result in Figure 17b. The results show that the modified BA-NOSB PD model can accurately capture the crack propagation paths of the Al_2_O_3_ ceramic plate under normal impact, demonstrating the ability to model impact problems and the reliability of damage prediction. In particular, the established model does not take into account the asymmetric factors that may exist in the experiment such as the projectile deflection and the microscopic defects of the sample. Therefore, the PD simulation result does not show the asymmetry of the crack path in the experiment.

**Figure 18:**
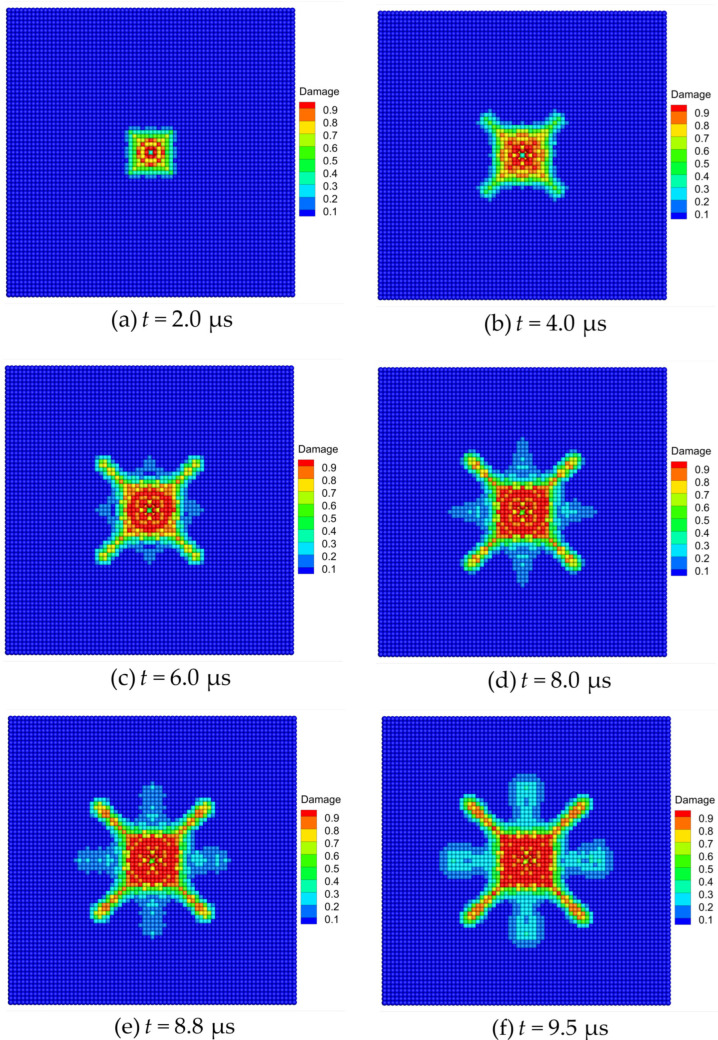
Damage evolution patterns of the Al_2_O_3_ ceramic plate under normal impact.

The damage evolution and crack growth of the Al_2_O_3_ ceramic plate during normal impact are shown in Figure 18. With the impact of the steel ball on the ceramic plate, the compression stress waves generated in the center of the target constantly spread to the boundary surfaces. The compression stress waves are reflected as the tensile stress waves once they have propagated to a free surface. When t=2.0 μs, the upper surface of the target forms a square damage zone. Two circumferential cracks appear in the central damage zone, and cracks initiate outward at the boundary of the damage zone, as shown in Figure 18a. With the continuous propagation, reflection, and overlapping of stress waves, the square damage zone on the target surface expands further, and a collapse is observed in the central area of the target. In addition, four radial cracks along the directions of 45°, 135°, 225°, and 315° begin to form, as shown at the instant of t=4.0 μs in Figure 18b. When t=6.0 μs, an obvious circular crack forms in the center of the target, and four radial cracks along the directions of 0°, 90°, 180°, and 270° initiate and grow outward, as seen in Figure 18c. Under the continuous penetration of the steel projectile, the crack tips along the directions of 0°, 90°, 180°, and 270° grow outward, and then obvious radial cracks form, as shown at the instant of t=8.0 μs in Figure 18d. When t=8.8 μs, four radial cracks form on the upper surface of the target along the directions of 0°, 90°, 180°, and 270°, as shown in Figure 18e. Finally, the damage tends to be stable at t=9.5 μs, while the center of the target produces fracture behaviors such as fragmentation and collapse, and the top surface produces eight radial cracks along the 0°, 45°, 90°, 135°, 180°, 225°, 270°, and 315° directions, as shown in Figure 18f.

## 4. Conclusions

In this work, a novel bond-associated non-ordinary state-based peridynamic model for quasi-brittle materials is proposed. The JH2 constitutive model is implemented in the framework of the bond-associated non-ordinary state-based peridynamic theory in the first place, to describe the mechanical behavior of quasi-brittle materials. A new calculation scheme of the volumetric strain in the constitutive model is proposed with the introduction of the bond-associated deformation gradient to improve stability and accuracy in describing the bulking effect. A new treatment strategy for broken bonds is proposed as well. In addition, considering the effect of tensile-shear deformation on material damage, a universal tensile-shear coupling bond-breaking criterion is proposed and implemented in the bond-associated non-ordinary state-based peridynamics.

The effectiveness and robustness of the modified bond-associated non-ordinary state-based peridynamic model are verified to some extent by two benchmark examples. The edge-on impact and normal impact experiments on ceramics are numerically modeled and simulated. The results reveal that the proposed model can effectively control zero-energy mode and eliminate numerical oscillations. Compared with the other four control schemes listed in this work, the modified bond-associated model has a more stable zero-energy mode control capability and convenience. In addition, from the energy conservation analysis of the system, one can find better accuracy and robustness from the modified bond-associated non-ordinary state-based peridynamic model. Good agreement can be found with the experimental data in the two demonstration benchmark tests, which implies that the modified bond-associated non-ordinary state-based peridynamic model can accurately reproduce the fracture patterns and crack growth paths of ceramics in the experiment.

## Figures and Tables

**Table 1:** JH2 constitutive model parameters of the Al_2_O_3_ ceramic [56].

Parameter	Value	Parameter	Value
Density ρ (kg/m^3^)	3700	T (GPa)	0.2
Shear modulus G (GPa)	90.16	$P_{\mathrm{HEL}}$ (GPa)	1.46
Poisson’s ratio ν	0.22	$\sigma_{\mathrm{HEL}}$ (GPa)	2.0
A	0.93	$D_{1}$	0.005
B	0.31	$D_{2}$	1.0
C	0.0	$K_{1}$ (GPa)	130.95
M	0.6	$K_{2}$ (GPa)	0.0
N	0.6	$K_{3}$ (GPa)	0.0
${\dot{\varepsilon }}_{0}$	1.0	β	1.0

**Table 2:** Solutions to modify the original BA-NOSB PD model.

Solution A	Solution B	Solution C
$\mu =\mu_{p}=\rho /\rho_{0}-1$	$\mu =\mu_{b}=1/J_{b}-1$	$\mu =\mu_{b}=1/J_{b}-1$
${\underline{Y}}_{B}\langle \xi \rangle =\xi$	${\underline{Y}}_{B}\langle \xi \rangle =\xi$	${\underline{Y}}_{B}\langle \xi \rangle =\xi +\eta_{B}$

## Data Availability

The data used to support the findings of this study are available upon request from the corresponding author.
